# Advanced Material Strategies for Next-Generation Additive Manufacturing

**DOI:** 10.3390/ma11010166

**Published:** 2018-01-22

**Authors:** Jinke Chang, Jiankang He, Mao Mao, Wenxing Zhou, Qi Lei, Xiao Li, Dichen Li, Chee-Kai Chua, Xin Zhao

**Affiliations:** 1 State Key Laboratory for Manufacturing Systems Engineering, Xi’an Jiaotong University, Xi’an 710049, China; cjkxjtu@stu.xjtu.edu.cn (J.C.); maomaosjs@outlook.com (M.M.); zhouman8@stu.xjtu.edu.cn (W.Z.); leiqi310@stu.xjtu.edu.cn (Q.L.); dcli@mail.xjtu.edu.cn (D.L.); 2 Department of Chemistry, Stanford University, Stanford, CA 94305, USA; xil@stanford.edu; 3 Singapore Centre for 3D Printing, School of Mechanical and Aerospace Engineering, Nanyang Technological University, 50 Nanyang Avenue, Singapore 639798, Singapore; MCKCHUA@ntu.edu.sg; 4 Interdisciplinary Division of Biomedical Engineering, the Hong Kong Polytechnic University, Hung Hom, Kowloon, Hong Kong 999077, China; xin.zhao@polyu.edu.hk

**Keywords:** additive manufacturing, micro-/nano-scale 3D printing, bioprinting, 4D printing, conductive materials, biomaterials, smart materials

## Abstract

Additive manufacturing (AM) has drawn tremendous attention in various fields. In recent years, great efforts have been made to develop novel additive manufacturing processes such as micro-/nano-scale 3D printing, bioprinting, and 4D printing for the fabrication of complex 3D structures with high resolution, living components, and multimaterials. The development of advanced functional materials is important for the implementation of these novel additive manufacturing processes. Here, a state-of-the-art review on advanced material strategies for novel additive manufacturing processes is provided, mainly including conductive materials, biomaterials, and smart materials. The advantages, limitations, and future perspectives of these materials for additive manufacturing are discussed. It is believed that the innovations of material strategies in parallel with the evolution of additive manufacturing processes will provide numerous possibilities for the fabrication of complex smart constructs with multiple functions, which will significantly widen the application fields of next-generation additive manufacturing.

## 1. Introduction

Additive manufacturing (AM) has drawn tremendous attention from both academia and industry with its potential applications in various fields, such as electronics [1], sensors [2], microfluidics [3], and tissue engineering [4]. Unlike conventional subtractive manufacturing approaches, the AM process enables the fabrication of 3D macro/microstructures by adding materials in a layer-by-layer manner [5,6,7]. Conventional AM processes such as material extrusion [8] and powder bed fusion [9,10] cannot meet the increasing demands for the 3D fabrication of high-resolution features, living constructs, and smart structures. Various novel AM processes such as micro-/nano-scale 3D printing, bioprinting, and 4D printing have been developed as next-generation AM processes to fabricate complex 3D features with high resolution, in multimaterials, or with multifunctionalities. The development of advanced functional materials is important for the implementation of novel AM processes, which has exhibited great potential for the fabrication of 3D structures with multiple functions. For instance, the incorporation of conductive nanomaterials into high-resolution AM processes has significantly simplified the microfabrication processes for microscale electronic devices [11]. The combination of biologically relevant hydrogels and living components with AM has been proven to be an effective approach to fabricating 3D living tissues or organs with multiple cell types and biomimetic micro/nanoarchitectures [12]. In addition, the marriage of smart materials with AM has created a new research field of 4D printing [13]. Although the existing explorations are still at their early stages, these advanced material strategies for these next-generation AM processes will definitely accelerate innovation in various fields.

Here, a state-of-the-art review on advanced material strategies for novel AM processes is provided, which mainly includes conductive materials for micro-/nano-scale 3D printing, biomaterials for next-generation bioprinting, and smart materials for 4D printing. The advantages, limitations, and future perspectives for each area are discussed.

## 2. Conductive Materials for Micro-/Nano-Scale 3D Printing

Conductive features play important roles in modern electronic devices such as electrodes, sensors, flexible electronics, and microbatteries [14,15,16]. With the increasing demands for high performance and multiple functionalities, 3D conductive features were sorely needed, posing great challenges to conventional micro-fabrication techniques. Micro-/nano-scale 3D printing might provide an alternative and promising way to fabricate 3D complex conductive features based on conductive materials in an efficient and low-cost way [17]. Micro-/nano-scale 3D printing techniques used for the fabrication of conductive features mainly include material jetting, material extrusion [18], and electrohydrodynamic (EHD) printing [19]. Different materials and their composites were developed for AM to fabricate conductive features. These conductive materials could be mainly categorized into metal-based materials [20] and other conductive materials [21,22].

### 2.1. Advanced Metal-Based Materials for Micro-/Nano-Scale 3D Printing

Additive-manufactured micro-/nano-scale structures derived from metal-based materials exhibit excellent electrical conductivity. They are ideal materials for the fabrication of electrodes, connectors, and conductors. The metal-based materials for micro-/nano-scale 3D printing can be further classified into three groups: liquid metals, metal nanoparticles, and in-situ reactive metal inks.

**Figure 1:**
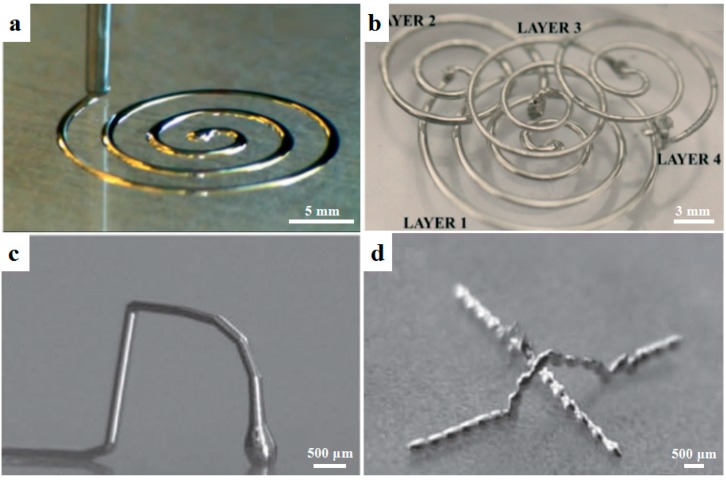
Liquid metals for microscale conductive features. (**a**) Direct printing of EGaIn liquid metal, a eutectic alloy of gallium (Ga) and indium (In) with a melting point of 15.5 °C; (**b**) 3D microfluidic channels with 3D-printed liquid metal as the sacrificial template, reprinted from [24] with the permission of John Wiley and Sons, Copyright 2013; (**c**,**d**) 3D conductive features directly printed from liquid metals, reprinted from [25] with the permission of John Wiley and Sons, Copyright 2013.

Liquid metals have recently attracted attention for the additive manufacturing of microscale conductive features due to their low melting temperature as well as their excellent conductivity. Liquid metals can be used for micro-/nano-scale 3D printing techniques such as direct writing and inkjet printing. Among various liquid metals, gallium-based liquid metal has a low melting temperature of 15~16 °C and exhibits a negligible vapor pressure as well as rheological and wetting properties [23]. For example, Parekh et al. [24] used a material extrusion process to print a eutectic alloy of gallium (Ga) and indium (In) (EGaIn) into 2D and 3D conductive structures at room temperature. The printed features could be further used as sacrificial templates to form 3D metal microfluidic channels, as shown in Figure 1a,b. Ladd et al. [25] employed a material extrusion process to fabricate freestanding wires, fibers, and interconnects based on the low-viscosity liquid metal EGaIn as shown in Figure 1c,d. The metal features were mechanically robust due to the formation of a thin oxide layer on the surface. The smallest feature size was about 10 μm and the stretchable interconnects could be incorporated into flexible substrates. Although liquid metals exhibited great potential in terms of printability and conductivity, novel liquid metals were urgently needed with low cost and excellent mechanical properties for the micro-/nano-scale 3D printing of complex conductive features.

**Figure 2:**
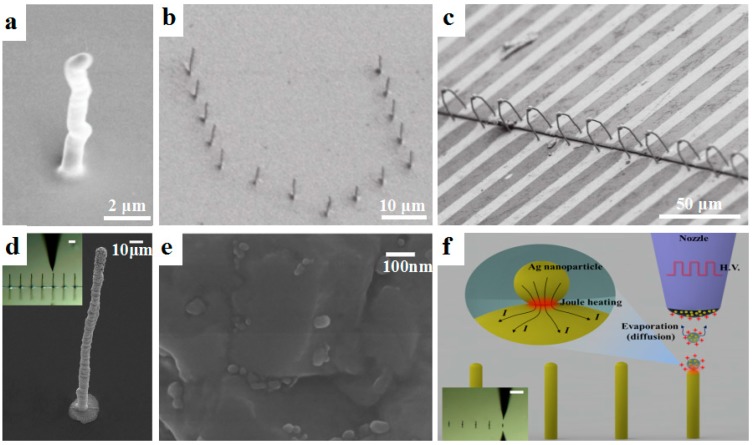
Metal nanoparticles for the micro-/nano-scale 3D printing of conductive features. (**a**) Ag pillar printed by electrohydrodynamic 3D printing; (**b**) A “U-shaped” array of Cu; (**c**) “Bridge-like” Ag interconnects used as electrical connection of the two electrodes, reprinted from [19] with the permission of John Wiley and Sons, Copyright 2015; (**d**) SEM image of an electrohydrodynamically (EHD) printed sub-10 μm 3D pillar with high aspect ratio over 35. The inset is a photograph of the EHD printing process, and the scale bar is 30 μm; (**e**) The magnified surface morphology of the Ag nanoparticles on the 3D pillar structure; (**f**) Schematic of spontaneous nanoscale Joule heating in the fabrication of the high-aspect-ratio 3D structures for which the electrohydrodynamic 3D printing technique was employed, reprinted from [30] with the permission of John Wiley and Sons, Copyright 2017.

The most frequently used materials for additive manufacturing of micro-/nano-scale conductive structures are the composite inks containing metal nanoparticles. Currently, various metal nanoparticles such as gold nanoparticles [26], silver nanoparticles [27], and copper nanoparticles [28] have been synthesized for micro-/nano-scale 3D printing. For example, Skylar-Scott et al. [29] employed silver nanoparticle inks to print freestanding 3D metallic architectures, such as interconnects and springs, based on directed energy deposition. The printed silver wires had a large range of feature diameter from 1 μm to 200 μm and the smallest electrical resistivity of 5.4 × 10^−6^ Ω·cm. An et al. [19] used silver and copper nanoparticle mixed inks to fabricate various conductive features such as high-aspect-ratio pillars, freestanding walls, helical structures, and arch-like bridges based on EHD printing (Figure 2a,b). The 3D-printed conductive bridge was 37 μm in length and 1.7 μm in diameter, exhibited a resistance of 6 Ω after annealing at 150 °C, and could electrically connect two separate electrodes as shown in Figure 2c. In another study of EHD 3D printing of metal nanoparticles, Zhang et al. [30] employed EHD printing to fabricate 3D submicroscale silver structures with a high aspect ratio of 35, as shown in Figure 2d. The Ag nanoparticles are shown in Figure 2e. Unlike conventional methods to achieve high conductivity through post-sintering, nanoscale joule heating at the interface between the charged Ag nanoparticles was used during the EHD printing process (Figure 2f). These 3D-printed features based on metal nanoparticles exhibited high conductivity close to that of bulk metal. However, relatively high temperature treatments such as laser-based annealing, thermal annealing, and joule hearing (>200 °C) were commonly required [31], which significantly limited their applications in the additive manufacturing of conductive features on flexible polymeric substrates.

**Figure 3:**
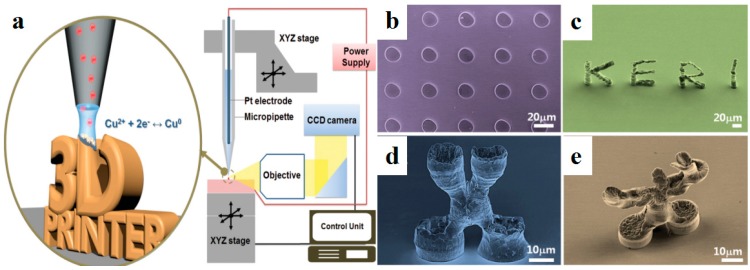
Electrodeposition-based 3D printing of Cu microarchitectures with in situ reactive inks. (**a**) Schematic illustration of electrodeposition-based 3D printing of in situ reactive Cu ions; (**b**–**e**) 3D-printed Cu architectures with different shapes, reprinted from [34] with the permission of John Wiley and Sons, Copyright 2015.

To overcome the above-mentioned problems, in situ reactive metal inks were developed as promising materials for the micro-/nano-scale 3D printing of high-resolution conductive features. Walker et al. [32] demonstrated in situ reactive silver inks that could fabricate highly conductive silver features (>10^4^ S/cm) based on a material extrusion process. Upon heating at a mild temperature of 90 °C, the printed features exhibited excellent conductivity equivalent to that of bulk silver (6.25 × 10^−5^ S/cm). Choi et al. [33] further developed novel silver inks that could react at room temperature. A microreactor-assisted printing method was used to print silver features that showed an electrical conductivity of half that of the bulk silver materials. Seol et al. [34] used an electrodeposition-based printing process to print 3D solid structures. As shown in Figure 3a, the Cu ions near the substrate were important for the morphology and density of printed structures. Freestanding 3D microscale Cu architectures were printed as shown in Figure 3b–e.

### 2.2. Other Conductive Materials for Micro-/Nano-Scale 3D Printing

**Figure 4:**
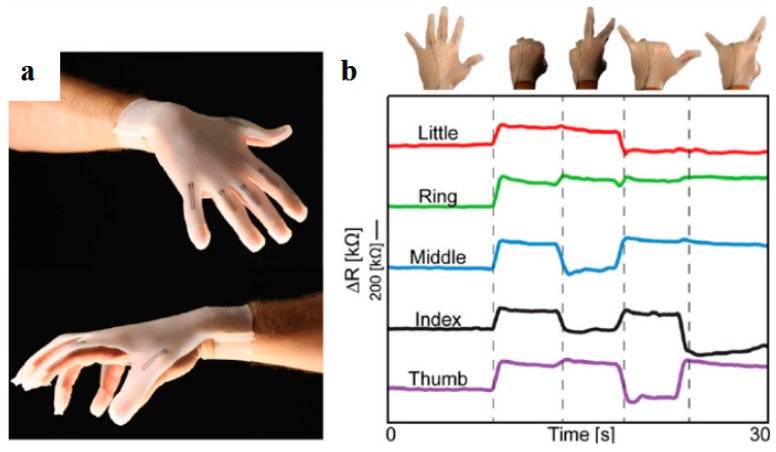
Microscale 3D-printed functional devices with conductive materials. (**a**,**b**) A glove with 3D-printed carbon-based conductive lines and its resistance response of each finger during flexing, reprinted from [36] with the permission of John Wiley and Sons, Copyright 2013.

Besides metal-based materials, other conductive materials such as carbon-based materials, lithium ions, and zinc have been used for the AM of high-resolution conductive features. As an important type of conductive materials, carbon-based materials such as carbon black, carbon nanotubes (CNTs), carbon nanofiber, and graphene have been added into polymeric materials due to their excellent conductive and sensing abilities [31]. For instance, carbon black particle inks were used to fabricate flexible strain sensors and piezoresistive sensors by material extrusion, which could embed functional sensors and electronics in a single and low-cost process [35]. Muth et al. [36] used carbon conductive grease, an inexpensive suspension of carbon black particles, to print highly stretchable sensors within an elastomeric matrix. The embedded sensors could sense the change of resistance at different hand positions as shown in Figure 4a,b. Zhang et al. [37] mixed reduced graphene oxide with polylactic acid (PLA) to print stretchable 3D conductive structures, which achieved a very high conductivity of 600 S/cm. CNTs were also printed on papers that could maintain their electrical connectivity after repetitive folding and unfolding cycles. Jeong et al. [38] used a polymer blend comprising polystyrene sulfonate (PSS) and multiwall carbon nanotubes (MWCNTs) to prepare EHD printing inks with different properties. The printed MWCNT/PSS lines with minimum width of 89 μm displayed a maximum conductivity of 39.3 S/cm and were used as source/drain electrodes in highly reliable organic field-effect transistors (OFETs). The carbon-based conductive materials were used as functional additives in micro-/nano-scale 3D printing techniques. However, the conductivity and stability of the additive-manufactured structures from carbon-based materials were susceptible due to the use of insulating polymers and dispersity of carbon-based materials.

Lithium ions and zinc are other types of conductive materials used for the fabrication of microbatteries. Compared with conventional rechargeable batteries, additive-manufactured microbatteries could potentially increase energy density by efficient use of the limited 3D space. For example, Sun et al. [39] printed 3D interdigitated microbattery architectures composed by Li_4_Ti_5_O_12_ (LTO) and LiFePO_4_ (LFP) material extrusion, which served as the anode and cathode materials, respectively. The results indicated that the printed 3D microbatteries with minimum width of 30 μm possessed a high areal energy density of 9.7 J/cm^2^ and a power density of 2.7 mW/cm^2^. However, due to the absence of conducting additives, the electrodes possessed much lower electrical conductivity which significantly limited electrochemical performance. Fu et al. [40] further added graphene into the LEP cathode and LTO anode materials. The extrusion-based microbatteries exhibited very stable cycling performance with specific capacities. Hu et al. [41] coated carbon on the surface of LiMn_1−x_Fe_x_PO_4_ nanocrystals for the printing of lithium-ion batteries which showed enhanced electric conductivity and high capacity with impressive electrochemical performance. In comparison with lithium-ion batteries, zinc–silver batteries have demonstrated high specific energies up to 300 Wh/kg, and are able to offer a high power density of over 600 W/kg [42]. Ho et al. [43] fabricated arrays of pillars from alkaline zinc–silver materials by using material jetting and the printed electrodes demonstrate enhanced capacity.

## 3. Biomaterials for Next-Generation Bioprinting

Biomaterials are widely used to mimic the extracellular matrix for cell and tissue cultures in the fields of tissue engineering, biomedicine, organ-on-a-chip, and drug-release devices [44,45,46]. Additive manufacturing of biomaterials, called bioprinting, has been upgraded to meet the demands for complex 3D functional living tissues with spatial control of the microenvironment, generated using multiple ingredients [47,48,49].

Some biomaterials have been widely used in bioprinting, such as bioceramics and metallic biomaterials. For example, calcium phosphate biomaterials including hydroxyapatite (HA) [50], tricalcium phosphate (TCP) [51], bioglass (including bioglass 45S5) [52], and bioactive glasses 13–93 [53] have excellent biocompatibility and compositional similarity with bone and teeth; consequently, they have been used in the production of porous scaffolds for bone and tissue regeneration. Metallic biomaterials like titanium-based materials are printed using the powder bed fusion process with the minimized pore size of 400 μm [10]. These common additive-manufactured biomaterials will not be discussed in this review. Here, three categories of biomaterials with great potential for next-generation AM are summarized, including biopolymers for the high-resolution bioprinting of tissue engineering scaffolds, hydrogels for cell printing, and multimaterial bioprinting.

### 3.1. Biopolymers for High-Resolution Bioprinting of Tissue Engineering Scaffolds

**Figure 5:**
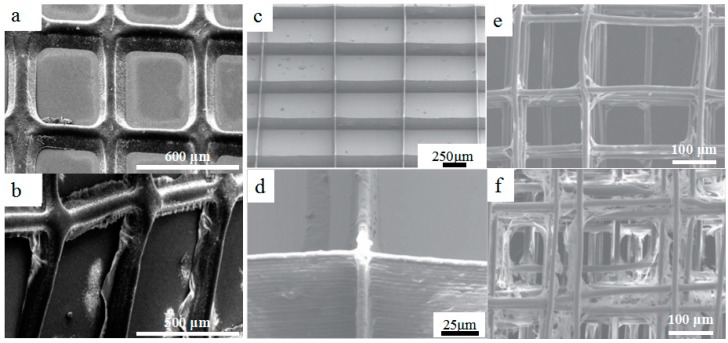
3D printed biopolymer with micro-/nano-scale structures. (**a**,**b**) Electrohydrodynamically printed drug-loaded polycaprolactone (PCL) polymer patches and drug-loaded PCL–polyvinyl pyrrolidone (PVP) patches after 90 mins drug release, reprinted from [48] with the permission of Nature Publishing Group, Copyright 2017; (**c**,**d**) SEM images of electrohydrodynamically printed polyethylene oxide (PEO)–PCL scaffolds with multiwall carbon nanotube (MWCNT) content of 0.5 *w/v*%, reprinted from [55] with the permission of IOP Publishing, Copyright 2017; (**e**,**f**) SEM characterizations of melt electrohydrodynamic 3D-printed cell scaffold constructs cultured for 7 days; (**e**) SEM images of cell morphology in the scaffolds with a fiber spacing of 250 μm; (**d**) SEM images of cell morphology in the scaffolds with a fiber spacing of 100 μm, reprinted from [56] with the permission of IOP Publishing, Copyright 2017.

Biopolymers have been widely used in AM, owing to their advantages of good manufacturability and biological properties [54]. Recent progress in micro-/nano-scale 3D bioprinting has opened new ways to pattern high-resolution features with biopolymers. Various biopolymers, including polylactic acid (PLA), polycaprolactone (PCL), poly-L-lactide (PLLA), and their composites, have been used to build synthetic tissue engineering scaffolds via electrohydrodynamic (EHD) printing to provide a biomimetic microenvironment for cellular attachment, proliferation, and differentiation, since the printed fibers possess a suitable scale (about 800 nm–40 μm), very close to the scale of living cells. For example, Wang et al. [48] employed an EHD printing technique to fabricate aligned-fiber antibiotic patches with a mean diameter of printed fiber ranging from 12.5 ± 1.2 to 7.5 ± 0.9 μm by using polycaprolactone (PCL) and polyvinyl pyrrolidone (PVP). As shown in Figure 5a,b, a distinct difference in high-resolution patch morphology was observed between the drug-PCL and drug-PCL/PVP systems. Drug release from the pure PCL patches was comparatively slow when compared with the PCL/PVP system. The release behavior could be influenced by the void size of patches achieved through the EHD printing technique together with the printing material and dosage forms.

Our group developed microfibrous scaffolds based on polyethylene oxide (PEO), PCL, and multiwall carbon nanotubes (MWCNTs) with a printed fiber diameter of about 10 μm by employing solvent-based electrohydrodynamic 3D bioprinting, as shown in Figure 5c,d. Significant cell proliferation was found on the PEO-PCL-MWCNT composite scaffolds, showing that MWCNTs facilitated cell elongation and alignment along with the microfibers [55]. Pure PCL could also be used in melt electrohydrodynamic 3D printing to produce filaments with a size of about 10 μm which could be precisely stacked into 3D walls, as shown in Figure 5e,f [56]. Hydroxyapatite nanoparticles were also added into PCL to print high-resolution scaffolds for bone tissue engineering, mimicking the collagen fibers and HA crystals in natural bone. The microscale composite scaffolds exhibited good biocompatibility and were beneficial to cell proliferation and alignment in vitro [57,58]. All these results showed that the composite biomaterials for 3D bioprinting could potentially be used to regulate cell behavior and growth and facilitate tissue regeneration on multiscale and multimaterial levels. Scaffolds fabricated using 3D EHD bioprinting had good control over fiber arrangements and appropriate internal pores, which could enhance cellular ingrowths and were able to regenerate complex large tissues with curved geometries and microscale fibrous structures.

### 3.2. Hydrogels for 3D Bioprinting and Cell Printing

**Figure 6:**
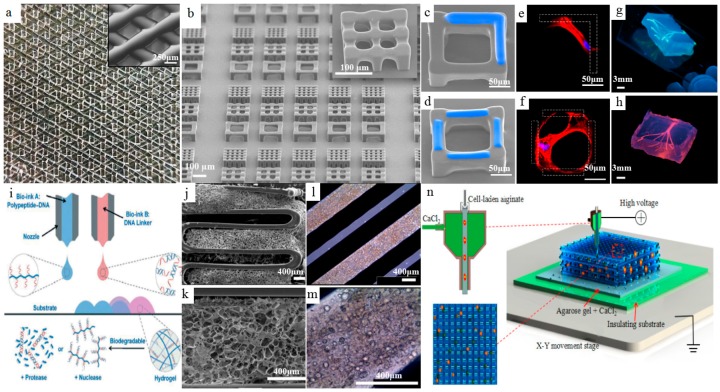
Bioprinted hydrogels. (**a**) Macroscopic view of additive-manufactured five-layer gelatin. Inset: Magnification of scaffold porosity, reprinted from [61] with the permission of Nature Publishing Group, Copyright 2017; (**b**) SEM images of 3D cubic microscaffolds via vat photopolymerization process. (**c**,**d**) SEM images of the cubic microscaffolds with in situ printed GelMA (blue); (**e**,**f**) the fluorescent staining of f-actin (red) and nuclei (blue) of the human mesenchymal stem cells (hMSCs) cultured in the corresponding microscaffolds (gelation shown as the dashed area), reprinted from [62] with the permission of Nature Publishing Group, Copyright 2017; (**g**) Photographs of the bioprinted agarose templates (green); (**h**) Respective microchannels perfused with a fluorescent microbead suspension (pink, diameter of microchannels: 500 μm), reprinted from [63] with the permission of Royal Society of Chemistry, Copyright 2014. (**i**) Scheme of 3D bioprinting of the polypeptide–DNA hydrogel, reprinted from [66] with the permission of John Wiley and Sons, Copyright 2017; (**j**,**k**) SEM images of hybrid structure of cartilage decellularized extracellular matrix (cdECM) with PCL framework; (**l**,**m**) microscopic images of cell-printed structure of adipose decellularized extracellular matrix (adECM) with PCL framework, reprinted from [69] with the permission of Nature Publishing Group, Copyright 2014; (**n**) Schematic of coaxial nozzle-assisted electrohydrodynamic cell printing, reprinted from [73] with the permission of WHIOCE publishing, Copyright 2017.

Hydrogels are a class of three-dimensional (3D) networks formed by hydrophilic polymer chains embedded in a water-rich environment, such as methacrylate gelatin (GelMA), collagen, and alginate [59]. Through combination with hydrogels of different cross-link types, 3D bioprinting provides unprecedented control over hydrogel-based scaffolds with both biomimetic microenvironments and biological functions [60]. Laronda et al. [61] fabricated scaffolds that restored ovarian function with line width of about 100 μm using partially crosslinked, thermally regulated gelatin via an extrusion-based bioprinting technique, as shown in Figure 6a. They investigated how scaffold pore geometry affected the growth and maturation of ovarian murine follicles. The results showed that specific scaffold architectures achieved by the 3D bioprinting technique resulted in optimal murine follicle survival and differentiation in vitro. Xia et al. [62] fabricated arrays of 3D cubic microscaffolds with cubical size matching the single-cell size using a polymer material via a vat photopolymerization process (Figure 6b). As shown in Figure 6c–f, GelMA was in situ printed on the microscaffold. Cells specifically adhered to the GelMA-deposited areas but not the naked SU-8 (bioinert photoresist) surface. Bertassoni et al. [63] fabricated microchannel networks with various architectural features within photocrosslinkable hydrogel constructs by utilizing bioprinted agarose template fibers (Figure 6g,h). This newly developed 3D micromolding technique had been shown to be an effective technique for the vascularization of hydrogel constructs with useful applications in tissue engineering and organ-on-a-chip.

Hydrogel blends were further developed to improve their bioactivity for functional expression or suitability during a 3D bioprinting process. Stratesteffen et al. [64] utilized the tailored hydrogel blends of photocrosslinkable GelMA and type I collagen for a material extrusion process. The GelMA–collagen hydrogels exhibited favorable biological and rheological properties which were suitable for the formation of a capillary-like network. In this printing material system, the addition of collagen into GelMA led to enhanced cell spreading and a shear thinning behavior of the hydrogel solution, and did not alter the characteristic crosslinking time for GelMA. Almeida et al. [65] developed a scaffold with a line width of 75 μm using chitosan cross-linked with pectin which showed a higher degree of mineralization, swelling ratio, faster drug release, higher stiffness, and lower degradation rate in saline solution than did scaffolds fabricated using pure chitosan. Supramolecular polypeptide–DNA was also used to fabricate 3D hydrogels [66]. The printed structures were geometrically uniform without boundaries and could keep their shapes up to the millimeter scale without collapse due to their healing properties and high mechanical strengths. As shown in Figure 6i, the DNA sequences of bio-ink A (blue) and bio-ink B (red) were complementary, and hybridization caused crosslinking, leading to hydrogel formation (pink). The formed hydrogels were responsive to both proteases and nucleases, resulting in the full on-demand degradation of the hydrogel networks after printing.

Traditionally, cells were seeded into hydrogel scaffolds which could not possibly mimic the actual anatomical composition [12]. In recent years, cell printing technology, which allowed 3D living tissues or organs to be manufactured directly based on layer-by-layer deposition of cell hydrogels, was developed as a novel type of AM process for the next generation of manufacturing [67,68]. On one hand, some new cell hydrogels were used in cell printing for better imitation of the natural microenvironment. For example, Pati et al. [69] utilized novel cell-laden decellularized extracellular matrix (dECM) gels for the printing of cell-laden constructs capable of providing an optimized microenvironment conducive to the growth of three-dimensional structured tissue. This cell printing method enabled the printing of cell-laden dECM structures with high cell viability and functionality. Particular tissue constructs were printed with cell-laden dECM gels as shown in Figure 6j–m. On the other hand, some typical hydrogel materials like collagen, polyvinyl alcohol, and sodium alginate could be mixed with living cells as the printing ink for high-resolution cell printing [70]. For example, Shinjiro et al. [71] employed electrohydrodynamic 3D bioprinting to print alginate droplets with an average diameter ranging from several micrometers to tens of micrometers. The printed droplets were gelled instantly once deposited onto the CaCl_2_ coated substrates and a 3D biological structure could be stacked by controlling the movement of the print-head. Our group developed an electrohydrodynamic cell printing strategy with microscale resolution (<100 μm) and high cellular viability (>95%). Compared with the existing electrohydrodynamic cell jetting or printing explorations, an insulating substrate was used as the collecting surface which significantly reduced the electrical current in the electrohydrodynamic printing process from milliamperes (>0.5 mA) to microamperes (<10 μA). Moreover, the nozzle-to-collector distance was fixed as small as 100 μm, which benefited filament deposition control and stacking into 3D [72]. In another study by our group, a novel coaxial nozzle-assisted electrohydrodynamic cell printing strategy was developed to fabricate living 3D cell-laden constructs with filament dimension about 80 μm and cell viability over 90%, as shown in Figure 6n [73].

### 3.3. Multimaterial Bioprinting

Multimaterial bioprinting with a high degree of spatial and compositional precision is able to create complex and organized 3D microscale structures composed of various cell types, ECMs, and many other elements, and achieve the complexity and biofunctional performance of tissue constructs. For example, bioprinting of several materials including hydrogels and embedded filaments would increase the mechanical stability of the constructs. Kundu et al. [74] managed to print cells directly by encapsulating cells into alginate hydrogel and fabricating scaffolds with the cell-encapsulated alginate hydrogel and PCL using an AM process. Results of in vitro and in vivo experiments showed higher ECM formation and enhanced cartilage tissue and type II collagen fibril formation in the PCL–alginate gel hybrid scaffold. Jin-Hyung Shim et al. [75] fabricated a mechanically enhanced additive-manufactured construct with a line width of 200 μm using PCL and hydrogels via a multihead tissue/organ building system. PCL, which showed relatively higher mechanical properties than hydrogel, was used to enhance the mechanical stability of the constructs. Visser et al. [76] reinforced soft hydrogels with highly organized, high-porosity microfiber networks that were fabricated with melt EHD printing. The stiffness of the gel/scaffold composites increased synergistically, compared with hydrogels or microfiber scaffolds alone. The stiffness and elasticity of the composites approached that of articular cartilage tissues. The reinforcement was applicable to numerous hydrogels which offered a fundament for producing tissue constructs with biological and mechanical compatibility.

Through multimaterial printing, multiple cell types, biomaterials, and other components of the microfluidic device were successfully printed with precise and reproducible spatial control for various organ-on-a-chip applications, which would promote full mimicry of the natural conditions of the organs. Recently, Lind et al. [77] designed six functional inks based on piezoresistive, high-conductance, and bioavailable soft materials and printed them at the microscale in a single continuous procedure to characterize microarchitectures that guided the self-assembly of physio-mimetic laminar cardiac tissues. A new class of cardiac microphysiological chips embedded with soft sensors was developed through multimaterial bioprinting to provide a noninvasive, continuous electronic readout of the contractile stress of multiple laminar cardiac microtissues. Compared with the previous biomimetic microsystems which were not well suited for higher-throughput or longer-term studies [78,79,80], the novel system of six materials-based bioprinted instrumented cardiac microphysiological devices would drastically simplify data acquisition and leverage the ability to track the temporal development in tissue mechanics, enabling new insights into tissue development and drug-induced structural and functional remodeling.

## 4. Smart Materials for High-Resolution 4D Printing

Smart materials are defined as stimulus-responsive materials that change their shape or functional properties under certain stimuli such as temperature, solvent, pH, electricity, light, and so on [81,82,83]. The combination of AM with smart materials has created a promising research area known as 4D printing [84,85]. Compared with conventional AM processes, 4D printing offers novel possibilities to fabricate dynamic or smart structures and devices instead of stationary ones. Although it is a newly emerging technique, extensive explorations have been made in various fields. In this section, smart materials for high-resolution 4D printing are reviewed, which mainly include shape-shifting materials and piezoelectric materials.

### 4.1. Shape-Shifting Materials for High-Resolution 4D Printing 

**Figure 7:**
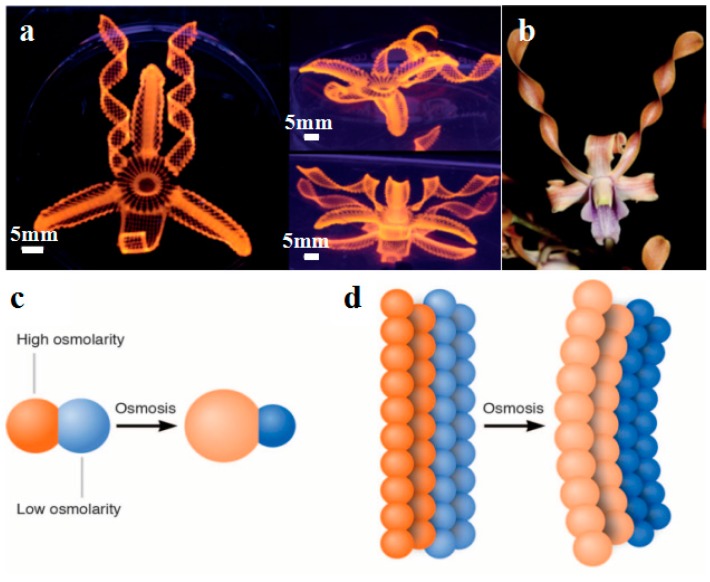

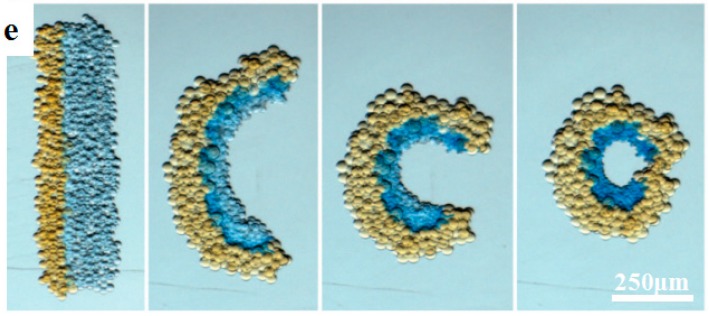
Shape-shifting structures fabricated by 4D printing. (**a**,**b**) Biomimic shape-shifting flowers, reprinted from [87] with the permission of Nature Publishing Group, Copyright 2016; (**c**) Schematic of two droplets of different osmolarities joined by a lipid bilayer. The flow of water through the bilayer causes the droplets to swell or shrink; (**d**) Schematic of a droplet network that comprises two strips of droplets of different osmolarities. The transfer of water between the droplets induces an overall deformation of the network; (**e**) Photographs of a rectangular network folding into a circle over about 3 h, reprinted from [88] with the permission of The American Association for the Advancement of Science, Copyright 2013.

High-resolution smart structures fabricated by 4D printing from shape-shifting materials can change their shapes or properties with time or space, which makes it possible to precisely control microscopic shape changes and provides novel tools for potential applications in soft microrobotics, biomedical engineering, microscale biomimetics, and drug delivery. For example, Ge et al. [86] prepared lamina composites by printing glassy fibers within an elastomeric matrix. Glassy fibers with a size of 32–64 μm exhibited shape-shifting properties when the temperature was over their glass transition temperature. The printed 3D structures can be thermomechanically programmed to different complex configurations. Gladman et al. [87] demonstrated a novel biomimetic 4D printing technique by the extrusion of a programmable material solution which contained clay, monomer, nanofibrillated cellulose (NFC), photoinitiator, enzyme/glucose, and deionized water. The printed flower structures with the smallest filament size of about 100 μm could be controlled to change their shapes as shown in Figure 7a,b. This programmable shape-shifting behavior was mainly caused by the differences in the swelling ratios of active and rigid materials under water. Villar et al. [88] employed high-resolution material jetting to print two kinds of materials with different osmolarity properties (Figure 7c). The printed straight lines have the smallest diameter of 50 μm, which can be further transformed into a circular structure as shown in Figure 7e. The shape-shifting is enabled by the osmolality gradient of the KCl droplets in the printed structures (Figure 7d).

**Figure 8:**
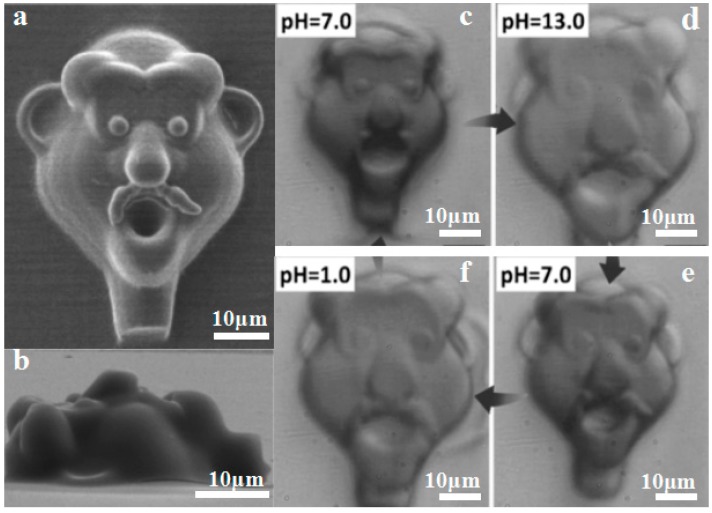

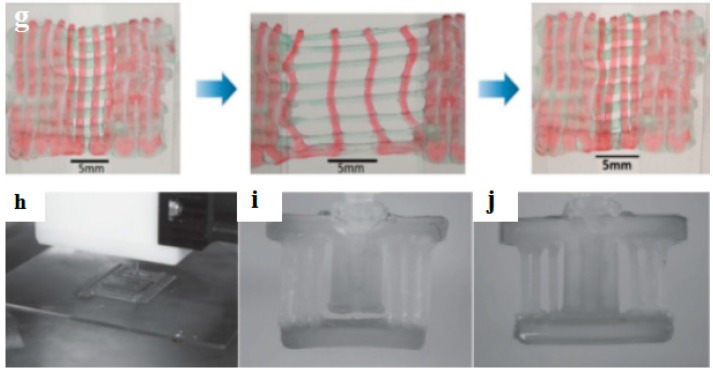
Shape-shifting proteins and hydrogels fabricated by 4D printing (**a**,**b**) A microscale face fabricated by femtosecond laser direct writing (FsLDW) of protein (**c**,**e**) the printed face changed in shape due to stimulation of pH (Scale bar 10 μm), reprinted from [89] with the permission of John Wiley and Sons, Copyright 2011; (**g**) 4D-printed microscale shape memory hydrogels recovered from stretching, reprinted from [91] with the permission of John Wiley and Sons, Copyright 2015; (**h**,**j**) 4D-printed shape-shifting hydrogels for micro actuating, reprinted from [92] with the permission of John Wiley and Sons, Copyright 2015.

Shape-shifting proteins were recently developed for high-resolution 4D printing, owing to their characteristics in response to chemical signals. For instance, Sun et al. [89] made photocrosslinked protein with bovine serum albumin (BSA) and a photosensitizer methylene blue, and used it to fabricate micro-/nano-scale architectures by directed energy deposition. The pH-responsive materials could be printed into protein microlenses with diameter from 1 μm to 100 μm, exhibiting rapid and reversible swelling-to-shrinking behavior. As shown in Figure 8a–f, a 3D relief portrait of a face was printed and swelled to about 150% when the pH value was increased from 7 to 13. Lee et al. [90] used two-photon photolithography to print 3D BSA–protein hydrogel microstructures ranging from 10 to 30 µm with programmable shape changes. By spatially controlling the cross-linking density of BSA at a nanometer length scale, directional responsiveness of printed 3D microstructures was achieved with high precision.

Other kinds of shape-shifting materials such as shape memory hydrogels have also been explored for high-resolution 4D printing. Printed hydrogel structures exhibit high toughness and stretchability similar to natural tissues, which can withstand physiological mechanical loads. Hong et al. [91] employed a high-resolution 4D printing technique to fabricate highly deformable and tough structures from a PEG-Alginate-Nanoclay hydrogel. The printed structures contained an interpenetrating network that could be stretched to 300% of its original length and subsequently recover to its initial shape, as shown in Figure 8g. Shannon et al. [92] printed 250 μm thick ionic covalent entanglement (ICE) hydrogels with mechanically robust and thermally actuating properties (Figure 8h–j). By modifying the amount of thermally responsive poly(N-isopropylacrylamide) (PNIPAAm) network in the hydrogels, the gels showed reversible length changes of 41–49%.

### 4.2. Piezoelectric Materials for High-Resolution 4D Printing of Smart Devices 

**Figure 9:**
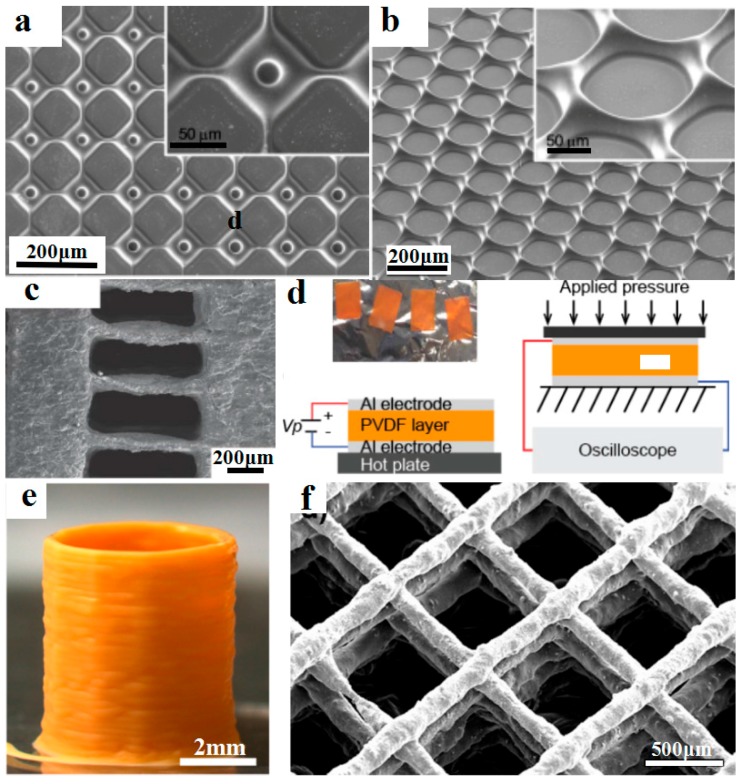
4D-printed smart piezoelectric microstructures. (**a**,**b**) Different microstructures printed by digital projection printing (DPP), including (**a**) square arrays and (**b**) honeycomb arrays, reprinted from [93] with the permission of American Chemical Society, Copyright 2014; (**c**,**d**) Printed using projection microstereolithography (PμSL) technique for an all-polymer-based piezoelectric device, reprinted from [94] with the permission of Elsevier, Copyright 2017; (**e**) One-step 4D-printed piezoelectric cylinder; (**f**) SEM image of inclined top view of a 3D spanning 9-layer piezoelectric scaffold, reprinted from [95] with the permission of American Chemical Society, Copyright 2017.

Piezoelectric materials, which can convert the energy of external stresses into electric energy or vice versa, have been widely used for the high-resolution 4D printing of smart devices such as energy harvesting, acoustic imaging, electrical actuators, and microsensors. For example, Kim et al. [93] employed microscale digital projection printing (DPP) to fabricate smart 3D microstructures with piezoelectric polymers, incorporating barium titanate (BaTiO_3_, BTO) nanoparticles into photoliable polymer solutions, as shown in Figure 9a,b. By adding 10 wt·% of BTO nanoparticles, micro/nanostructures with smallest size about 1 μm gained good mechanical properties and exhibited a piezoelectric coefficient (d_33_) of around 40 pC/N. The presented method could be potentially used to fabricate smart micro-/nano-scale structures for potential applications to bioengineering, materials science, physics, and chemistry.

Polyvinylidene fluoride (PVDF) is an important type of piezoelectric material and has been used for the high-resolution 4D printing of smart piezoelectric devices. For example, Chen et al. [94] developed a photocurable piezoelectric resin for projection microstereolithography (PμSL) as shown in Figure 9c,d. Diethyl fumarate (DEF) was used as a solvent to dissolve PVDF for better manufacturability and piezoelectric characteristics. The optimized inks contain 35 wt·% PVDF and were successfully used to print 3D structures with high resolution up to 7.1 μm. The resultant piezoelectrically active film exhibited a piezoelectric voltage coefficient (g_33_) of 105.12 × 10^−3^ V∙m/N. Bodkhe et al. [95] used a vat photopolymerization method for the printing of smart 3D structures with high dielectric and piezoelectric properties. As shown in Figure 9e,f, composites of BaTiO_3_ nanoparticles and PVDF were printed into fibers with a size of about 56 ± 6 μm and stacked to build smart microsensors which demonstrated a maximum voltage output of 4 V upon finger tapping.

## 5. Conclusions and Future Perspectives

Recent advances in the development of various functional materials for novel AM processes have improved the accuracy of manufacturing and enhanced the functional complexities of the printed structures. The innovations in material strategies and AM processes should be emphasized in parallel to construct complex structures or devices with multiple characteristics such as electroconductibility, sensing, biocompatibility, and stimulus-responsiveness, which will extremely widen the application fields of next-generation 3D printing. Table 1 is used to summarize these developments for easier comparison.

Micro-/nano-scale 3D-printed architectures with conductive materials have exhibited great potential for future perspectives of electronics, sensors, and other conductive devices. On one hand, the metal-based additive-manufactured features often possess very high electrical conductivity values (5% to 100% conductivity of bulk materials), which is suitable for fabricating electrodes and conductive interconnects. Metal-nanoparticle inks have been widely explored for minimizing the size of printed features. However, the printing process commonly requires high-temperature postprocessing processes which are incompatible with flexible substrates such as polymers. Liquid metals and reactive inks might be alternative materials to address this issue. On the other hand, the printing of carbon-based materials, which are relatively low in conductivity, can be used to develop functional sensors and devices. Additive-manufactured microbatteries made with lithium ions or zinc have higher energy density and are promising in autonomously powered microelectronics and biomedical devices.

Future development of bioprinting materials would focus on various aspects, including printability, mechanical properties, biocompatibility, degradation, and biomimicry [75]. The material properties should be optimized according to the specific AM processes so that they can be deposited accurately with the desired spatial and temporal control. The maintenance of 3D structures, in resisting or producing specific forces, is essential for the biological functions of the constructs. Developing novel composite biomaterials with different ingredients to obtain reprogrammed mechanical properties and functionality may be a promising approach to fabricating various tissues and organs with different mechanical requirements. Moreover, the biomaterials should degrade with spatial and temporal controllability to meet the requirements of different regeneration stages in vivo. Exciting achievements have been made in precisely regulating the microstructural or physical microenvironments for cell growth by the printing of micro-/nano-scale biomimetic features or the addition of biomimetic components. One of the major challenges for cell or organ printing is to develop novel bio-inks that can not only be printed to maintain specific-tissue architectures but also facilitate the growth and proliferation of the embedded cells.

The development of smart materials should be in parallel with advances in 4D printing techniques. Using 4D printing techniques, smart materials can be fabricated into complex structures with specific responsiveness. Besides this, smart materials for high-resolution 4D printing might require physical or chemical modifications of specific additives such as rheology and viscosity modifiers, photo-initiator agents, crosslinking agents, or sacrificial agents. From this aspect, a key direction is the development of composite materials for 4D printing by combining functional smart materials with printable materials. The stimuli- and time-related dynamics will lead to the shape-shifting of high-resolution printed structures. The conceptualization of 4D printing has set in motion the discovery of more targets with broader functionalities of smart materials. As high-resolution 4D printing techniques rapidly evolve, micro-/nano-scale stimuli-responsive architecture together with multiscale design, multimaterial printing, and time-dependent function regulation will definitely find wide applications in various fields.

## Figures and Tables

**Table 1:** Summary of advanced material strategies for additive manufacturing.

Material Class	Materials		AM process	Applications
Conductive materials	Metal-based materials	Liquid metals, metal nanoparticles, In stu reactive metal inks.	Material jetting [26,43] Material extrusion [24,25,32,33,35,36,37,39,40,41] Directed energy deposition [29] EHD printing [19,27,28,30,34,38]	metal channels, interconnects, electrodes, electronics, sensors, microbatteries.
	Other conductive materials	carbon-based materials, lithium-ion zinc.		
Biomaterials	Biopolymers	PLA, PCL, PLLA, PEO, PVP.	EHD printing [48,55,56,57,58,67,76] Material extrusion [50,61,63,64,65,66,74] Vat photopolymerization [62] Cell printing [68,69,70,71,72,73]	bio-scaffolds, fabrication of tissues and organs, drug release, microfluidic devices, organ-on-a-chip applications.
	Hydrogels	Methacrylate gelatin (GelMA), collagen, alginate, hydrogel blends.		
Smart materials	Shape-shifting materials	shape-shifting glasses, shape-shifting solutions, shape-shifting proteins, shape-shifting hydrogels.	4D printing [84,85] Material extrusion [86,87,91,92] Material jetting [88] Directed energy deposition [89] two-photon photolithography [90] digital projection printing [93] projection micro-stereolithography [94] vat photopolymerization [95]	Soft microrobotics, Biomedical engineering, Drug delivery, Piezoelectric devices.
	Piezoelectric materials	Barium titanate (BaTiO_3_, BTO), Polyvinylidene fluoride (PVDF).		
